# Isolation of known and potentially pathogenic tick-borne microorganisms from European ixodid ticks using tick cell lines

**DOI:** 10.1016/j.ttbdis.2019.02.008

**Published:** 2019-04

**Authors:** Ana M. Palomar, Shonnette Premchand-Branker, Pilar Alberdi, Oxana A. Belova, Anna Moniuszko-Malinowska, Olaf Kahl, Lesley Bell-Sakyi

**Affiliations:** a Centre of Rickettsiosis and Arthropod-Borne Diseases, CIBIR, C/ Piqueras, 98, Logroño 26006, La Rioja, Spain; b The Pirbright Institute, Ash Road, Pirbright, Woking, Surrey GU24 0NF, UK; c Department of Biology, University of York, Wentworth Way, York YO10 5DD, UK; d The Roslin Institute and Royal (Dick) School of Veterinary Studies, University of Edinburgh, Easter Bush, Midlothian EH25 9RG, UK; e Chumakov Institute of Poliomyelitis and Viral Encephalitides (Chumakov FSC R&D IBP RAS), prem. 8, k.17, pos. Institut Poliomyelita, poselenie Moskovskiy, Moscow 108819, Russia; f Martsinovsky Institute of Medical Parasitology, Tropical and Vector Borne Diseases, Sechenov University, 20-1 Malaya Pirogovskaya St., Moscow 119435, Russia; g Department of Infectious Diseases and Neuroinfections, Medical University in Białystok, Zurawia 14, 15-540 Białystok, Poland; h Tick-radar GmbH, 10555 Berlin, Germany; i Department of Infection Biology, Institute of Infection and Global Health, University of Liverpool, Liverpool Science Park IC2, 146 Brownlow Hill, Liverpool L3 5RF, UK

**Keywords:** Tick cell line, *Ixodes*, *Dermacentor*, *Rickettsia*, *Spiroplasma*, *Mycobacterium*

## Abstract

Ticks harbour and, in many cases transmit to their vertebrate hosts, a wide variety of pathogenic, apathogenic and endosymbiotic microorganisms. Recent molecular analyses have greatly increased the range of bacterial species potentially associated with ticks, but in most cases cannot distinguish between surface contaminants, microorganisms present in the remains of the previous blood meal and truly intracellular or tissue-associated bacteria. Here we demonstrate how tick cell lines, primary cell cultures and organ cultures can be used to isolate and propagate bacteria from within embryonic and adult *Ixodes ricinus*, *Dermacentor marginatus* and *Dermacentor reticulatus* ticks originating from different parts of Europe. We isolated and partially characterised four new strains of *Spiroplasma* from The Netherlands, Spain and Poland, two new strains of *Rickettsia raoultii* from Russia and Poland, one strain of *Rickettsia slovaca* from Spain and a species of *Mycobacterium* from the UK. Comparison with published sequences showed that the *Spiroplasma* strains were closely related to *Spiroplasma ixodetis* and the *Mycobacterium* isolate belonged to the *Mycobacterium chelonae* complex, while the *R. raoultii* and *R. slovaca* strains were similar to previously-validated species.

## 1 Introduction

Ixodid ticks of the genera *Ixodes* and *Dermacentor* are the most widespread and important vector species infesting livestock and humans in Western, Northern, Central and Eastern Europe (Estrada-Peña et al., 2006; Medlock et al., 2013; Rubel et al., 2016). They transmit a broad range of viral, bacterial, protozoan and helminth pathogens of veterinary and/or medical importance (Heyman et al., 2010; Hubálek and Rudolf, 2012; Jongejan and Uilenberg, 2004; Otranto et al., 2013; Portillo et al., 2015; Socolovschi et al., 2009). In addition, they harbour a variety of bacteria of low or unknown pathogenicity including *Spiroplasma* spp., *Candidatus* Midichloria mitochondrii and some *Rickettsia*, *Coxiella* and *Francisella* spp. (Bonnet et al., 2017; Duron et al., 2017; Taylor et al., 2012), some of which may represent true endosymbionts (Duron et al., 2017). While numerous recent studies using molecular-based detection have highlighted the prevalence, distribution and expanding ranges of obligate intracellular bacteria in European *Ixodes* and *Dermacentor* ticks, fewer studies have actually isolated such microorganisms directly from ticks into vertebrate or arthropod cell culture, an essential prerequisite for their full characterisation (Alberdi et al., 2012a; Bell-Sakyi et al., 2015; Henning et al., 2006; Kurtti et al., 2015; Mediannikov et al., 2008, 2010, 2012, 2014; Novakova et al., 2016; Santibáñez et al., 2015; Simser et al., 2002; Wijnfeld et al., 2016).

Tick cell lines offer a useful and effective medium for isolation and propagation of tick-borne bacteria from tick tissues or homogenates (Bell-Sakyi et al., 2007, 2015, 2018; Mediannikov et al., 2012, 2014; Santibáñez et al., 2015; Simser et al., 2002; Wijnfeld et al., 2016). Bacteria can also be isolated from primary tick cell cultures (Alberdi et al., 2012a; Ferrari et al., 2013; Mattila et al., 2007; Simser et al., 2001). Thus tick cell culture can be used as a sensitive detector and multiplier of endosymbiotic bacteria that may be present in the host tick at levels too low for molecular detection techniques. Successful PCR amplification of bacterial DNA from infected ticks can be affected by insufficient bacterial DNA in comparison to host DNA, presence of inhibitors (Schrader et al., 2012) and limited sensitivity of the assays. Moreover, PCR assays cannot distinguish between genomic DNA of viable and non-viable bacteria present in the sample, whereas only viable bacteria will grow *in vitro*.

Here we report attempted tick cell culture isolation and propagation of tick-borne bacteria from *Ixodes ricinus* ticks from the United Kingdom, The Netherlands, Poland and Spain, *Dermacentor marginatus* ticks from Russia and Spain, and *Dermacentor reticulatus* ticks from The Netherlands, Russia, Germany and Poland. These comprised engorged female ticks whose eggs were used to generate primary cell cultures with a view to establishing novel cell lines, and unfed or partially-fed male and female ticks potentially harbouring microorganisms. Using a panel of susceptible tick cell lines, we successfully propagated isolates of *Spiroplasma* spp. from Dutch, Polish and Spanish ticks and *Rickettsia* spp. from Polish, Russian and Spanish ticks. In addition, we isolated a fast-growing *Mycobacterium* sp. from a British tick, demonstrating the applicability of tick cell culture techniques in confirming tick-bacteria associations only previously implied by molecular analysis.

## 2 Materials and methods

### 2.1 Ticks

**Fig. 1:**
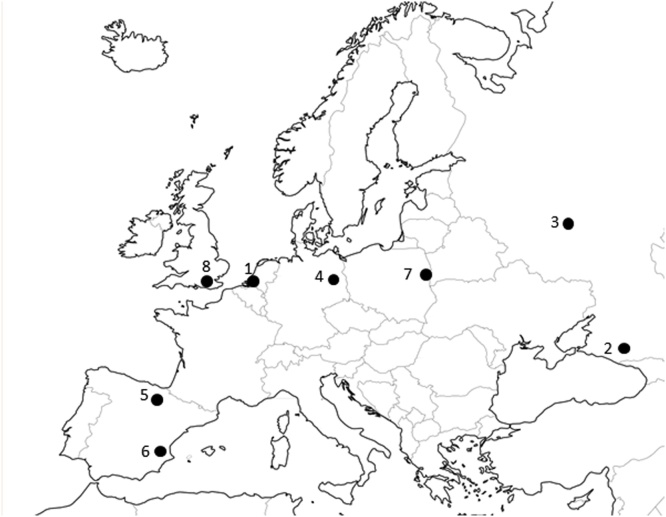
Location of sites of origin of ticks used in this study. 1. Zeeland, The Netherlands; 2. Karachay-Cherkess Republic, Russia; 3. Kaluga Region, Russia; 4. Berlin, Germany; 5. La Rioja, Spain; 6. Valencia, Spain; 7. Białystok and Białowieza, Poland; 8. Surrey, UK.

The locations of origin of the ticks used in this study are shown in Fig. 1. Fully-engorged female *I. ricinus* and *D. reticulatus* ticks were kindly provided by the Utrecht Centre for Tick-borne Diseases, Utrecht University, The Netherlands. The ticks had been collected as unfed adults at a field site in Zeeland, The Netherlands (Dintelse Gorzen, 51°37′N, 4°15′E; Fig. 1, site 1) and fed to repletion in the laboratory as previously described (Alberdi et al., 2012a; Nijhof et al., 2007). Fully-engorged adult female *D. marginatus* and *D. reticulatus* ticks were obtained from a colony of first-generation adults derived from eggs laid by female ticks collected from the field, maintained at the Chumakov Institute of Poliomyelitis and Viral Encephalitides, Moscow. The *D. marginatus* ticks were collected near Cherkessk in the Karachay-Cherkess Republic (44°18′N, 42°03′E; Fig. 1, site 2), while the *D. reticulatus* ticks originated from Visokinichi village in Zhukovsky district of the Kaluga region (54°54′N, 36°55′E; Fig. 1, site 3). Fully-engorged female *D. reticulatus* were collected from a domestic dog that had acquired them locally in Dallgow-Döberitz near Berlin, Germany (52°54′N, 13°05′E; Fig. 1, site 4) in October 2014. Fully-engorged female *I. ricinus* were collected from cattle in Tobía (42°17′N, 2°50′W; Fig. 1, site 5), La Rioja, Spain in September 2015. Fully-engorged female *D. marginatus* were collected from wildlife in Valencia, Spain (Fig. 1, site 6): one tick from an Iberian wild goat (*Capra pyrenaica*) in Cortes de Pallas (39°13′N, 0°57′W) in September 2015, and a second tick from a wild boar (*Sus scrofa*) in Llocnou de Sant Jeroni (38°54′N, 0°17′W) in February 2016. Russian and Spanish ticks were identified using taxonomic keys (Estrada-Peña et al., 2004, 2014; Filippova, 1997; Manilla, 1998). All engorged female ticks were surface-sterilised by immersion for 5 min in 0.1% benzalkonium chloride, 1 min in 70% ethanol and two changes of sterile deionised water, allowed to dry on sterile filter paper and incubated singly in sterile 50 mm plastic Petri dishes at 28 °C, 100% relative humidity until oviposition was completed.

Unfed adult *D. reticulatus* of both sexes and a single unfed female *I. ricinus* were collected from vegetation in September 2014 at two field sites in eastern Poland (Fig. 1, site 7) about 20 km south-west of Białystok (52°58′N, 23°05′E) and on the southern edge of the Białowieza National Park (52°42′N, 23°52′E) and stored at 15 °C, 100% relative humidity for 9 months before processing. Partially-fed *I. ricinus* adults of both sexes removed from a dog that had acquired them locally in Surrey, UK (51°17′N, 0°38′ W; Fig. 1, site 8), between May and August 2015 (n = 11) and May and June 2016 (n = 12) were kindly provided by The Pirbright Institute.

### 2.2 Preparation of primary tick cell cultures

Primary cell cultures were set up from eggs laid by the engorged female ticks when the rectal sacs of the developing embryos were visible. Briefly, the eggs were surface-sterilised by immersion for 1 min in 70% ethanol followed by two rinses in Hanks’ balanced salt solution (HBSS). The eggshells were then crushed with the flattened end of a glass rod in HBSS to release the embryos, the resultant tissue suspension was filtered through plastic gauze with 300 μm pore size and centrifuged at 200 × *g* for 5 min, and the tissue pellet was resuspended in 2.2 ml of complete culture medium with antibiotics (100 units/ml penicillin and 100 μg/ml streptomycin). Complete culture media used included L-15, H-Lac, L-15B and combinations thereof (Bell-Sakyi, 2004). The tissue suspension was incubated in a sealed, flat-sided culture tube (Nunc) in ambient air at 28 °C; medium was changed weekly by removal and replacement of ½–¾ of the medium volume.

### 2.3 Inoculation of tick cell lines with tick organs

A panel of continuous or putative tick cell lines derived from *I. ricinus*, *Ixodes scapularis*, *Dermacentor albipictus*, *D. marginatus*, *Dermacentor nitens*, *Rhipicephalus appendiculatus* and *Rhipicephalus microplus* were used in attempts to isolate and propagate bacteria (Table 1). For isolation from adult tick organs, unfed and partially-fed adult ticks were surface-sterilised as described above for fully-engorged ticks, allowed to dry, embedded dorsal side uppermost in sterile histological wax and dissected under HBSS. The dorsal integument was removed by cutting round the midline with a scalpel, and the internal organs were removed and inoculated into a flat-sided tube of cells of a tick cell line. The combined cell and organ cultures were incubated in ambient air at 28 °C and medium (with antibiotics as above) was changed weekly by removal and replacement of ¾ of the medium volume. For isolation from primary tick embryo-derived cell cultures, aliquots of culture supernate were inoculated directly into cell lines derived from the same tick species or inoculated after centrifugation at 1500 × *g* for 5 min into cell lines derived from a different species. When bacteria were detected in primary cell cultures, adult tick organ cultures and subcultures, aliquots of culture supernate were centrifuged as above to remove intact cells and inoculated into cultures of established or putative cell lines (Table 1).

**Table 1:** Tick cell lines used for isolation and propagation of tick-borne bacteria.

Cell line	Parent tick species	Culture medium^a^ and incubation temperature	Reference
ANE58	*Dermacentor nitens*	L-15B300; 32 °C	Kurtti et al., 1983
BME/CTVM2	*Rhipicephalus microplus*	L-15; 28 °C	Bell-Sakyi, 2004
BME/CTVM23	*Rhipicephalus microplus*	L-15; 32 °C	Alberdi et al., 2012a
BME/PIBB36	*Rhipicephalus microplus*	L-15; 28 °C	Bell-Sakyi et al., 2018
DALBE3	*Dermacentor albipictus*	L-15B300; 32 °C	Policastro et al., 1997
DMAR8T^b^	*Dermacentor marginatus*	L-15B; 28 °C	This study
IDE8	*Ixodes scapularis*	L-15B; 32 °C	Munderloh et al., 1994
IRE/CTVM19	*Ixodes ricinus*	L-15; 28 °C	Bell-Sakyi et al., 2007
IRE/CTVM20	*Ixodes ricinus*	L-15/L-15B; 28 °C	Bell-Sakyi et al., 2007
IRE11	*Ixodes ricinus*	L-15B300; 32 °C	Simser et al., 2002
ISE6	*Ixodes scapularis*	L-15B300; 32 °C	Kurtti et al., 1996
RA243	*Rhipicephalus appendiculatus*	L-15; 32 °C	Varma et al., 1975

a Complete culture media as described previously (Bell-Sakyi, 2004; Munderloh et al., 1999).

b A putative cell line derived from embryonic *D. marginatus* ticks kindly provided by the Chumakov Institute of Poliomyelitis and Viral Encephalitides, Moscow, Russia; the cells were successfully cured of *Rickettsia raoultii* infection by two successive treatments with tetracycline, but were later lost to fungal contamination after being used in the present study.

### 2.4 Monitoring cell and organ cultures by light and electron microscopy

Primary cell cultures and cell lines inoculated with adult tick organs were examined weekly by inverted microscope for cell growth and presence of cytopathic effect (CPE). Giemsa-stained cytocentrifuge smears, prepared when CPE was detected (primary cell cultures) or at 2–8 week intervals (organ cultures) from culture supernate (50–100 μl) or whole resuspended cell cultures (50 μl) as described previously (Alberdi et al., 2012a), were examined under oil immersion at ×500 and ×1000 magnification (Leitz Orthoplan) for presence of bacteria. Samples of cell lines into which bacteria had been subcultured were processed for transmission electron microscopy and visualised as described previously (Alberdi et al., 2012a).

### 2.5 Cryopreservation of infected cultures

Tick cell cultures in which bacteria were detected by microscopy and/or PCR were resuspended by pipetting and held on ice. Dimethyl sulphoxide was added to give a final concentration of 10%, the cell suspension was mixed gently and dispensed immediately into ice-cold labelled cryovials which were rapidly frozen in dry ice and transferred to the vapour phase of a liquid nitrogen storage tank.

### 2.6 DNA extraction and PCR

Samples of culture supernate and whole resuspended cultures were centrifuged at 13,000 × *g* for 10 min at room temperature. DNA was extracted from the resultant pellets using a DNeasy blood and tissue kit (Qiagen), following the manufacturer’s instructions for Gram-negative bacteria.

DNA extracts were screened for detection of bacterial species using a pan-bacterial PCR assay that amplifies a 1,500-bp fragment of the 16S rRNA gene (Weisburg et al., 1991; Table 2). The samples that yielded positive results with the pan-bacterial PCR were also analysed for presence of bacteria using genus-specific PCR assays. Selection of these assays was based on the sequences obtained from the 16S rRNA gene PCR products and/or the detection of a microorganism in a culture using light microscopy ± presence of CPE. Specifically, the gene fragments targeted for bacterial identification are listed in Table 2: for *Spiroplasma* spp. the 16S-23S rRNA intergenic transcribed spacer (ITS), the RNA polymerase beta subunit (*rpoB*) and the 16S rRNA (16S rRNA); for *Rickettsia* spp. the 120-kDa protein antigen (*ompB*), the PS120 protein (*sca4*) and the 190-kDa protein (*ompA*); for *Mycobacterium* spp. the 65-kDa heat shock protein (*hsp65*), the superoxide dismutase (*sodA*) and the RNA polymerase beta subunit (*rpoB*). These PCRs were carried out as described by the respective authors (Table 2). Furthermore, a PCR for the amplification of the 17-kDa lipoprotein gene (17-kDa) of *Francisella* spp. was designed; the primer set was based on specific *Francisella*-like endosymbiont sequences available in GenBank (accession nos: from AY375408 to AY375414). The specificity of this PCR was verified in silico using BLASTN analysis in GenBank. In addition, two specific PCR assays for the identification of microorganisms of the domain Archaea, that amplify two different fragments of the 16S rRNA gene, and two pan-fungal PCR assays that amplify the internal transcribed spacer (ITS) and the large subunit (LSU) of the rRNA gene (Table 2) were also used in this study. All the PCR primer pairs, their source references, sizes of the amplicons (bp) and annealing temperatures used in the assays are shown in Table 2.

**Table 2:** PCR primer pairs and conditions used in the study. F: Forward; R: Reverse; bp: base pairs; Tm: melting temperature; N = A/C/G/T; Y = C/T; K: G/T.

Organism	Target gene	Primer sequence (5’→ 3’)	Fragment size (bp)	Tm (ºC)	Reference
Pan-bacterial	16S rRNA	F:AGAGTTTGATCCTGGCTCAG	1500	60	Weisburg et al., 1991
		R:ACGGCTACCTTGTTACGACTT			
*Spiroplasma* spp.	ITS	F:GGTGAATACGTTCTCGGGTCTTGTACACAC	600–1000	60	Volokhov et al., 2006
		R:TNCTTTTCACCTTTCCCTCACGGTAC			
	*rpoB*	F:GGNTTTATTGAAACACCATAYGCTC	1443	63 Touchdown	Haselkorn et al., 2009
		R:GCATGTAATTTATCATCAACCATGTGTG		53	
	16S rRNA	F:AGAGTTTGATCCTGGCTCAG	˜500	55	Fukatsu and Nikoh, 2000
		R:TAGCCGTGGCTTTCTGGTAA			
*Rickettsia* spp.	*ompB*	F:AAACAATAATCAAGGTACTGT	811	55	Roux and Raoult, 2000
		R:TACTTCCGGTTACAGCAAAGT			
	*sca4*	F:ATGAGTAAAGACGGTAACCT	928	50	Sekeyova et al., 2001
		R:AAGCTATTGCGTCATCTCCG			
	*ompA* (semi-nested)	F:ATGGCGAATATTTCTCCAAAA	631	46	Roux et al., 1996
		R:GTTCCGTTAATGGCAGCATCT			Regnery et al., 1991
		F:ATGGCGAATATTTCTCCAAAA	532	48	
		R:AGTGCAGCATTCGCTCCCCCT			
*Mycobacterium* spp.	*hsp65*	F:ACCAACGATGGTGTGTCCAT	439	60	Telenti et al., 1993
		R:CTTGTCGAACCGCATACCCT			
	*sodA*	F:GAAGGAATCTCGTGGCTGAATAC	541	60	Adékambi and Drancourt, 2004
		R:AGTCGGCCTTGACGTTCTTGTAC			
	*rpoB*	F:GGCAAGGTCACCCCGAAGGG	764	64	Adékambi et al., 2003
		R:AGCGGCTGCTGGGTGATCATC			
*Francisella* spp.	17-kDa	F:GTAAGACATATAACTTACTTGT	408	50	This study
		R:AGCTAGTATCATGACACTTA			
Pan-archaeal	16S rRNA	F:ACKGCTCAGTAACACGT	806	63 Touchdown	Grosskopf et al., 1998
		R:GTGCTCCCCCGCCAATTCCT		52	Banning et al., 2005
	16S rRNA	F:TTCCGGTTGATCCCYGCCGGA	937	55	DeLong, 1992
		R:YCCGGCGTTGAMTCCAATT			
Pan-fungal	ITS	F: TCCGTAGGTGAACCTGCGG	570–590	62	White et al., 1990
		R: TCCTCCGCTTATTGATATGC			
	LSU	F: TCGATGAAGAACGCAGCG	1414	50	Vilgalys and Hester, 1990
		R: TACTACCACCAAGATCT			

A negative control containing water instead of template DNA was included in all PCRs. Where possible, positive control DNAs from microorganisms not commonly found in the tick species or geographical areas studied were used in the PCRs. *Borrelia spielmanii* DNA, kindly provided by Dr Volker Fingerle (German National Reference Centre for *Borrelia*) to the Centre of Rickettsiosis and Arthropod-Borne Diseases, was included in all the 16S rRNA pan-bacterial PCRs as a standard positive control. Positive controls were also included in some of the genus-specific PCRs for *Spiroplasma* spp. (DNA from *Spiroplasma* sp. strain Bratislava 1, Bell-Sakyi et al., 2015), *Rickettsia* spp. (DNA from *Rickettsia amblyommatis*; Santibáñez et al., 2017) and *Francisella* spp. (DNA from the tick cell line DALBE3, Alberdi et al., 2012b), and in the PCRs for Archaea (DNA from *Hyperthermus butylicus* kindly provided by Dr Thijs Ettema, Uppsala University, Sweden) and fungi (DNA from *Penicillium biourgeianum* kindly provided by the Centre of Rickettsiosis and Arthropod-borne Diseases, Spain).

### 2.7 Sequence analysis

Positive PCR products were purified using a High Pure PCR Product Purification kit (Roche Life Science) following the manufacturer’s instructions. Purified amplification products were sequenced in the forward and reverse directions, and homology searches were performed in the NCBI database using the BLAST search programme (http://blast.ncbi.nlm.nih.gov/Blast.cgi). Nucleotide sequences were aligned using the European Bioinformatics Institute multisequence software Clustal Omega (https://www.ebi.ac.uk/Tools/msa/clustalo/) for multiple sequence alignment. The resultant sequences that differed from those previously included in the NCBI database were submitted to GenBank using Sequin software (https://www.ncbi.nlm.nih.gov/Sequin/). Phylogenetic analyses were conducted using MEGA version 7 (www.megasoftware.net). The phylogenetic trees were constructed by the neighbour-joining method. Confidence values for individual branches of the resulting trees were determined by bootstrap analysis with 1000 replicates. The evolutionary distances were computed using the maximum composite likelihood method.

### 2.8 Next generation sequencing

Next Generation Sequencing (NGS) of a mixed bacterial isolate obtained from a British *I. ricinus* tick was carried out on an Illumina MiSeq platform in the Genomics and Bioinformatics Core Facility (CIBIR, Spain). The amplification of the hypervariable V3/V4 region of the 16S rRNA (550–580 bp) was performed using methodology previously optimised for the metagenome analysis of ticks. In brief, the library preparation was performed using the Illumina protocol “16S Metagenomic Sequencing Library Preparation”, the V3/V4 region was reconstructed according the Quantitative Insights Into Microbial Ecology (QIIME) protocol and the obtained Operational Taxonomic Units (OTUs) were compared using Greengenes database and refined using BLAST.

## 3 Results

In total, 36 primary embryo-derived cell cultures were screened for presence of bacteria (Table 3) as part of monitoring during attempted cell line establishment, a procedure that can take between one and seven years (Bell-Sakyi et al., 2018); if detected, attempts were made to isolate the bacteria into one or more tick cell lines. Similarly, organs from 31 adult ticks were inoculated into tick cell lines in an attempt to isolate bacteria (Table 3). All cultures were sampled for PCR analysis to detect and identify bacteria present therein. Positive PCR products were sequenced, compared with published data by BLAST analysis and novel sequences deposited in GenBank. The results are detailed in the following sections and summarised in Table 4.

**Table 3:** Numbers of primary tick cell cultures and adult tick organ cultures screened for presence of bacteria.

Type of culture	Tick species	Geographical origin	Number of cultures
Primary embryo-derived cell culture	*Ixodes ricinus*	The Netherlands	2
		Spain	9
	*Dermacentor marginatus*	Russia	10
		Spain	1
	*Dermacentor reticulatus*	The Netherlands	6
		Germany	7
		Russia	1
Adult organs co-cultivated with tick cell lines	*Ixodes ricinus*	UK	19
	*Ixodes ricinus*	Poland	1
	*Dermacentor marginatus*	Spain	1
	*Dermacentor reticulatus*	Poland	10

**Table 4:** Bacteria isolated into tick cell lines from *Ixodes ricinus*, *Dermacentor marginatus* and *Dermacentor reticulatus* ticks from six locations in Europe and Russia. bp: base pairs ID: inconclusive data ND: not done.

Organism	Isolated from tick (Country)	Isolated in cell line(s)	No of isolates^a^	Pan-bacterial 16S rRNA % identity (bp)- GenBank no	Species-specific genes					
					Gene	% identity (bp)-GenBank no	Gene	% identity (bp)-GenBank no	Gene	% identity (bp)-GenBank no
*Spiroplasma* sp.	*I. ricinus* (The Netherlands)	IRE/CTVM19, IRE/CTVM20, BME/CTVM2	2	100 (1335/1335)-KP967685	ITS	100 (813/813)-KP967686	*rpoB*	100 (1387/1387)-KP967687	16S rRNA	100 (453/453)-KP967685
	*D. marginatus* (Spain)	DMAR8, ANE58, BME/CTVM23, IRE/CTVM20	1	99.6-100^b^ (1339–1345/1345)-KP967685	ITS	99.6 (811/814)-KP967686	*rpoB*	99.8 (1391/1394)-KP967687	16S rRNA	100-99.6^b^ (448-446/448)-KP967685
	*D. reticulatus* (The Netherlands)	BME/CTVM2, BME/CTVM23, DALBE3	3	ID	ITS	99.6 (811/814)-KP967686	*rpoB*	99.8 (1355/1358)-KP967687	16S rRNA	99.6 (452/454)-KP967685
	*D. reticulatus* (Poland)	BME/CTVM23	1	ID	ITS	99.6 (811/814)-KP967686	*rpoB*	99.8 (1353/1356)-KP967687	16S rRNA	99.6 (451/453)-KP967685
*Rickettsia raoultii*	*D. reticulatus* (Poland)	BME/CTVM23	5	100 (1369/1369)-DQ365809^c^	*ompB*	100 (770/770)-JN242189	*sca4*	100 (849/849)-DQ365807	*ompA*	100 (587/587)-AH015609
	*D. marginatus* (Russia)	BME/CTVM2, BME/CTVM23	1	ND	*ompB*	100 (770/770)-JX683120	*sca4*	99.8 (868/870) JN242188	*ompA*	100 (488/488)-AH015609
*Rickettsia slovaca*	*D. marginatus* (Spain)	BME/CTVM23	1	100 (1368/1368)- CP002428	*ompB*	100 (770/770)- CP002428	*sca4*	100(859/859) CP002428	*ompA*	100 (586/586)- CP002428
*Mycobacterium* sp.	*I. ricinus* (UK)	BME/PIBB36	1	99.8 (1374/1377)-CP007220	*hsp65*	95.5 (383/401)-KM973026	*sodA*	94.5 (468/494)-AP018165	*rpoB*	95.4 (678/711)-KM392058

a Each isolate originated from an individual tick or egg batch laid by a single tick.

b >1 nucleotide different in a few positions.

c Only obtained from one of the isolates.

### 3.1 Isolation of bacteria from *I. ricinus* ticks

**Fig. 2:**
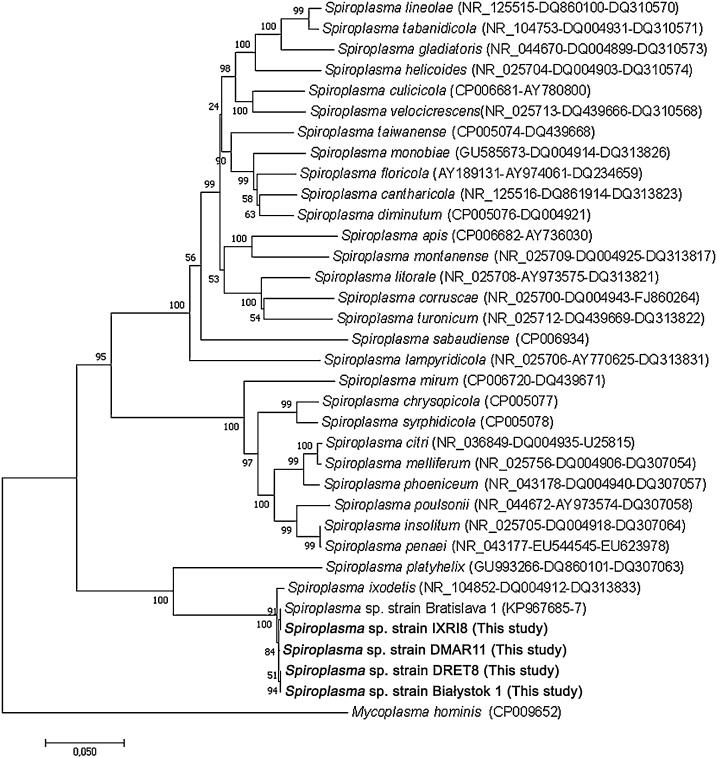
Unrooted dendrogram showing the phylogenetic position of *Spiroplasma* spp. isolated from *Ixodes ricinus* (strain IXRI8 from The Netherlands), *Dermacentor marginatus* (Strain DMAR11 from Spain) and *Dermacentor reticulatus* (strains DRET8 from Russia and Białystok 1 from Poland) in the present study (in bold), among valid *Spiroplasma* species. Phylogeny is inferred from comparison of 16S rRNA, ITS and *rpoB* (1993 positions in the final dataset) nucleotide sequences by the neighbour-joining method (1000 replicates). *Mycoplasma hominis* is used as outgroup. GenBank accession numbers of the genes used in the comparison are shown in brackets following each *Spiroplasma* species, with multiple accession numbers separated by dashes.

*Spiroplasma* were detected in Giemsa-stained smears prepared from two primary cell cultures set up from embryonic *I. ricinus* from The Netherlands, 7 months after culture initiation. Supernate from both primary cultures was passaged onto the *R. microplus* cell line BME/CTVM2 and the *I. ricinus* cell lines IRE/CTVM19 and IRE/CTVM20, and the two *Spiroplasma* isolates were maintained through 3–8 passages over a further 17 months before being cryopreserved. Both isolates caused CPE in all three cell lines; this occurred fastest in BME/CTVM2 cells. PCR analysis of DNA extracted from both isolates in all three cell lines gave positive results with *Spiroplasma*-specific primer pairs ITS, *rpoB* and 16S rRNA. The sequences obtained from the PCR products were identical to those deposited in GenBank from *Spiroplasma* sp. (Bratislava 1), a bacterium isolated from *I. ricinus* ticks from Slovakia (Table 4, Fig. 2).

**Fig. 3:**
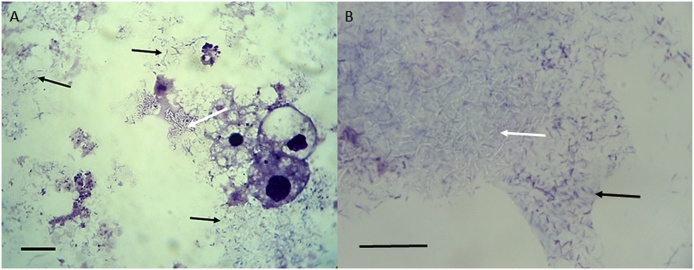
Microorganisms isolated from an *Ixodes ricinus* tick of UK origin. A *Mycobacterium* sp. belonging to the *Mycobacterium chelonae* complex (white arrows) and an unidentifiable filamentous putative microorganism that formed a biofilm (black arrows) were detected in a BME/PIBB36 culture inoculated 4 months previously with organs from a partially-fed female *I. ricinus* tick removed from a dog in Surrey, UK. Both microorganisms grew extracellularly in the presence of BME/PIBB36 cells (A) and axenically in complete L-15 medium (B). Giemsa-stained cytocentrifuge smears; scale bars = 10 μm.

**Fig. 4:**
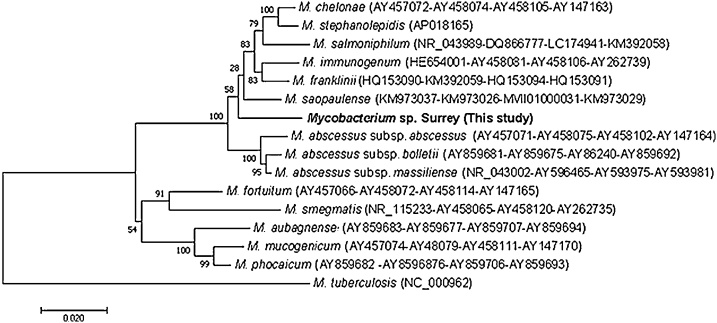
Unrooted dendrogram showing the phylogenetic position of *Mycobacterium* sp. Surrey (in bold), isolated in the present study, among valid *Mycoplasma* species included in the rapidly-growing group. Phylogeny is inferred from comparison of 16S rRNA, *hsp65*, so*dA* and *rpoB* (2809 positions in the final dataset) nucleotide sequences by the neighbour-joining method (1000 replicates). *Mycobacterium tuberculosis* is used as outgroup. GenBank accession numbers of the genes used in the comparison are shown in brackets following each *Mycobacterium* species, with multiple accession numbers separated by dashes.

Organs from one of the 16 partially-fed adult female *I. ricinus* removed from the UK dog and added to a *R. microplus* BME/PIBB36 culture yielded a mixed infection (Fig. 3) with a large, rod-shaped extracellular bacterium that did not stain with Giemsa and a small, filamentous, rod-shaped extracellular microorganism that grew predominantly in biofilm-like sheets (Fig. 3B). Both microorganisms were maintained through two passages in complete L-15 medium alone and in fresh BME/PIBB36 cultures before being cryopreserved. The mixed infection caused severe CPE in the tick cells after 4 months (initial infection) and 14 days (subcultures). 16S rRNA and pan-*Mycobacterium* (*hsp65*, *sodA* and *rpoB* genes) PCR assays revealed the presence of a fast-growing, free-living *Mycobacterium* sp. belonging to the *Mycobacterium chelonae* complex (Fig. 4, Table 4), which was presumed to be the identity of the large, rod-shaped bacteria that did not stain with Giemsa (Fig. 3). Sequences from the four amplified gene fragments were deposited in GenBank with accession numbers *Mycobacterium* sp. Surrey 16S rRNA (MG859279), *hsp65* (MG859273), *sodA* (MG859274), and *rpoB* (MG859276). Attempts to identify the smaller filamentous biofilm-forming microorganism using pan-bacterial 16S rRNA PCR were unsuccessful. Sequence data obtained from this assay only amplified the *Mycobacterium* isolate and did not indicate the presence of any other bacterium (duplicate DNA extracts obtained at different passage levels and from cultures with and without tick cells were analysed, and the PCR assay was repeated twice; the resultant sequences always corresponded to that of the *Mycobacterium* isolate). NGS was therefore applied to this culture. The technique showed 402,086 reads with a rarefaction curve that reached a plateau, so bacterial diversity had been satisfactorily detected in the sample. Almost all (99.95%) of the reads corresponded to those from the *Mycobacterium* sp. The remaining 0.05% reads showed homology with *Enterococcus* spp. and *Staphylococcus* spp., whose structure differs from that shown in the Giemsa-stained preparations of the biofilm-forming microorganism, and that were likely to represent contamination occurring during the DNA processing or an Illumina error during the sequencing run and multiplexing by the informatic process (Wright and Vetsigian, 2016). These results suggested that the latter microorganism was not a bacterium. In a further attempt at identification, two Archaea-specific and two fungal-specific PCR assays were performed, but all the PCRs yielded negative results for the culture sample, whilst the positive controls, DNA from *H. butylicus* and *P. biourgeianum* respectively, were amplified.

Bacteria were not isolated from any of the remaining *I. ricinus* from UK or from the Spanish and Polish *I. ricinus*. The pan-bacterial PCR (16S rRNA) gave positive results for 15/18 cultures inoculated with tick organs from UK (excluding the *Mycobacterium*-positive culture described above) and 9/9 Spanish *I. ricinus* primary cell cultures. Although some amplicons were not sequenced (faint bands) or the analysis of the sequences was inconclusive (possibly due to DNA from more than one microorganism being amplified), some of the sequences showed homology with *Candidatus* Midichloria mitochondrii. Specifically, 8/18 UK samples and 5/9 Spanish samples were positive for *Ca.* M. mitochondrii, but there was no evidence of bacterial replication. The resultant 16S rRNA sequences (between 648 and 1377 bp) were 100% identical to that of *Ca.* M. mitochondrii IricVA deposited in GenBank under the accession number CP002130.

### 3.2 Isolation of bacteria from *D. marginatus* ticks

Five pairs of primary cell cultures set up from individual egg batches laid by five Russian *D. marginatus* ticks were found to harbour a *Rickettsia* which was shown by PCR (primer sets for the amplification of *ompB*, *sca4* and *ompA* gene fragments; Table 4) to be *Rickettsia raoultii* at between 4.5 and 6.5 months post initiation. Isolates from one of each pair of cultures were made in BME/CTVM2 cells and cryopreserved 7 weeks later. All cultures were treated with 0.5 μg/ml tetracycline (Sigma) for 2 months, which apparently killed the *Rickettsia*; thereafter seven of the primary cultures died. *Rickettsia* reappeared in one of the three surviving primary cultures and its subcultures 26 months later; bacteria from this culture series were subinoculated into the *R. microplus* cell line BME/CTVM23 in which they grew vigorously and caused CPE, and species identity was confirmed by PCR (*ompB*, *sca4* and *ompA* gene fragments; Table 4) as *R. raoultii*. This isolate was designated *Rickettsia raoultii* (DMAR8) and its *sca4* sequence was deposited in GenBank with accession number MG859275. Subcultures derived from the primary culture were treated with tetracycline for 2 months and were negative for *Rickettsia* a year later; this putative cell line was designated DMAR8T and used in subsequent subinoculation experiments. The primary culture was left untreated and finally succumbed to the *R. raoultii* infection after a further 15 months.

One of two fully engorged female *D. marginatus* from Spain (Cortes de Pallas, Valencia) failed to lay any eggs, so her internal organs were dissected out and inoculated into a BME/CTVM23 cell culture. A fast-growing *Rickettsia* appeared within 6 weeks and was taken through one passage in BME/CTVM23 cells over the subsequent 6 months before being cryopreserved. The bacteria caused CPE in BME/CTVM23 cells but failed to infect IRE/CTVM19 cells as determined by examination of Giemsa-stained smears. PCR and sequence analysis using the three *Rickettsia*-specific PCR assays targeting the *ompB*, *sca4* and *ompA* genes (Table 4) revealed that the bacteria were *Rickettsia slovaca* with 100% identity to *R. slovaca* strain 13-B (Fournier et al., 2012). Our isolate was designated *R. slovaca* (Valencia).

A primary cell culture initiated from eggs laid by the other Spanish *D. marginatus* (from Llocnou de Sant Jeroni, Valencia) yielded a fast-growing *Spiroplasma*, which was subinoculated into DMAR8T, ANE58, IRE/CTVM20 and BME/CTVM23 cells before the primary culture was treated with 0.5 μg/ml tetracycline (Sigma) in an unsuccessful attempt to rescue it. The *Spiroplasma* grew prolifically in all four cell lines; in contrast to the parent *D. marginatus* primary culture, CPE was moderate in the heterologous tick cell lines and not seen in DMAR8T cells. Molecular characterisation of this isolate based on 16S rRNA, ITS and *rpoB* genes showed closest identity (99.6–100%) to *Spiroplasma* sp. strain Bratislava 1 isolated from *I. ricinus* (Table 4, Fig. 2). Sequences from three amplified gene fragments were deposited in GenBank with accession numbers *Spiroplasma* sp. DMAR11 ITS (MG859283), *rpoB* (MG859278) and 16S rRNA (MG859280).

### 3.3 Isolation of bacteria from *D. reticulatus* ticks

**Fig. 5:**
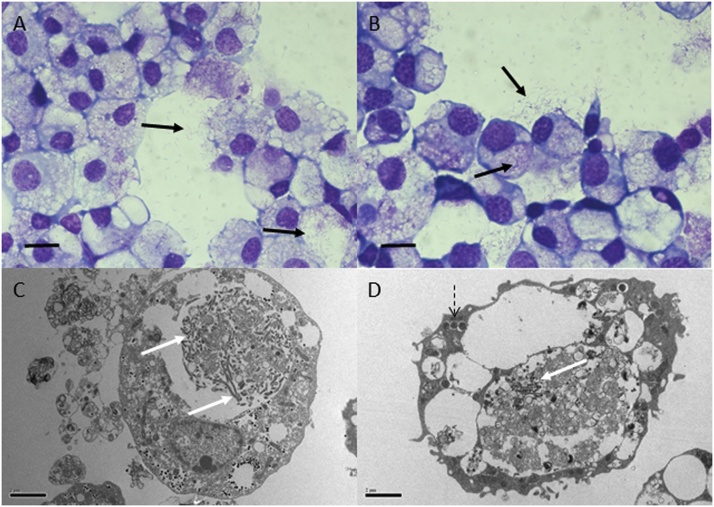
*Spiroplasma* sp. (solid black and white arrows) isolated from *Dermacentor reticulatus* primary cell cultures into DALBE3 (A, C), BME/CTVM2 (B) and BME/CTVM23 (D) cultures visualised in Giemsa-stained cytocentrifuge smears (A, B, scale bars = 10 μm) and transmission electron microscopy (C, D, scale bars = 2 μm). The BME/CTVM23 cell is also infected with *Rickettsia raoultii* bacteria (dotted arrow).

Isolation and propagation in tick cell lines of *R. raoultii* from the *D. reticulatus* from The Netherlands has already been reported (Alberdi et al., 2012a). Three of the six primary embryo-derived cell cultures reported by the previous authors were also infected with a *Spiroplasma* (Table 4). The sequences obtained corresponding to 16S rRNA, ITS and *rpoB* gene fragments showed maximum identity (99.6–99.8%) with *Spiroplasma* sp. strain Bratislava 1. Moreover, the ITS sequence was identical to that of the *Spiroplasma* isolated in the present study from the Spanish *D. marginatus* tick described above, but the 16S rRNA and *rpoB* sequences showed two and three nucleotide changes respectively between these two isolates (Table 4, Fig. 2). Representative sequences were deposited in GenBank with accession numbers *Spiroplasma* sp. DRET8 ITS (MG859284), *rpoB* (MG859277) and 16S rRNA (MG859282). Two of these *Spiroplasma* isolates were subinoculated into DALBE3, BME/CTVM2 and BME/CTVM23 cells (Alberdi et al., 2012a) and grew well as a mixed infection with *R. raoultii* in all three cell lines (Fig. 5). It was not possible to distinguish between possible CPE caused by the *Spiroplasma* and that caused by *R. raoultii*.

The primary embryo-derived cell culture set up from the Russian *D. reticulatus* did not yield any bacteria; PCR screening with pan-bacterial primers (16S rRNA) on multiple occasions over the succeeding 2–4 years gave negative results, and no bacteria were ever seen in Giemsa-stained cytocentrifuge smears.

Six of the ten cultures inoculated with internal organs from unfed adult *D. reticulatus* from Poland yielded bacteria: five isolates of *R. raoultii* (PCRs targeting 16S rRNA, *ompB*, *sca4* and *ompA* genes) from four female ticks and one male tick from Białystok, and one isolate of a *Spiroplasma* sp. from a male tick from Białystok (16S rRNA, ITS and *rpoB* sequences obtained), all in the cell line BME/CTVM23. No bacteria were isolated from three male and three female ticks collected in Białowieza and inoculated into BME/CTVM23, IRE/CTVM19, IDE8, ISE6 and RA243 cells. One of the *R. raoultii* isolates and the *Spiroplasma* isolate were successfully subcultured into BME/CTVM23 cells; all six bacterial isolates were cryopreserved after 8 months *in vitro*. The *ompA* sequence of the Polish *R. raoultii* isolate was identical to that obtained from the Russian *R. raoultii* isolate, but the corresponding *sca4* and *ompB* sequences showed three and one nucleotide changes respectively (Table 4).

Using the 16S rRNA pan-bacterial PCR, *Francisella* sp. DNA was detected in five of the ten tick cell cultures inoculated with internal organs of *D. reticulatus* from Poland and four of the seven embryo-derived primary cell cultures derived from eggs laid by engorged female *D. reticulatus* ticks from Germany. The resultant sequences (between 461 and 1377 bp) showed highest identity of 99.1% with a sequence from an *Ornithodoros moubata* symbiont (GenBank accession number AB001522), but were homologous to a shorter sequence from the *Francisella*-like endosymbiont of *D. reticulatus* strain HS249 (GenBank accession number JQ942365). All of these positive DNA extracts, in addition to those from the other five tick cell cultures inoculated with internal organs of Polish *D. reticulatus*, were positive in the specific 17-kDa *Francisella* PCR assay. The resultant sequences (380 bp) were identical to each other and homologous to that of the *Francisella*-like endosymbiont of *D. reticulatus* strain Kljajicevo (GenBank accession number HM629449). Representative sequences of this *Francisella*-like endosymbiont of *D. reticulatus* strain Bialowieza 1 were deposited in GenBank with accession number MG859281 (16S rRNA). No bacteria that could be identified as being *Francisella*-like were seen in any of the *D. reticulatus* primary cell cultures, or in any tick cell lines that received *D. reticulatus* organs.

## 4 Discussion

Successful *in vitro* isolation of tick-borne bacteria depends on having culture conditions suitable for supporting survival and growth of the microorganisms, whether they are intracellular or extracellular. The properties of tick cell cultures, namely that primary cell cultures may require many months of maintenance before significant cell growth commences and cell lines can be kept for long periods without subculture (Bell-Sakyi et al., 2018), make them particularly suitable for isolation of slow-growing or fastidious bacteria that may be present at very low levels in tick tissues. Antibiotics, as used in the present study to minimise environmental contamination of primary cell cultures and cell lines inoculated with tick organs, will obviously influence the spectrum of bacteria that can grow in such systems. Nevertheless, in the presence of penicillin and streptomycin we successfully propagated isolates of three different bacterial phyla, *Spiroplasma* (Tenericutes), *Rickettsia* (Proteobacteria) and *Mycobacterium* (Actinobacteria), from ticks of two different genera, *Ixodes* and *Dermacentor*, from multiple sites in Europe. Not surprisingly, spirochaetes, which are susceptible to penicillin, were not detected in any of the cultures, despite the prevalence of *Borrelia* spp. in *I. ricinus* ticks ranging from 0 to 19% in the UK and 0 to 32% in Spain (Barandika et al., 2008; Barral et al., 2002; Díaz et al., 2017; Hansford et al., 2017; Millins et al., 2016; Palomar et al., 2018; Toledo et al., 2009). Further studies are needed to develop protocols for successful isolation of bacteria susceptible to penicillin and/or streptomycin from European ticks. The absence of *Coxiella*-like endosymbionts, commonly found in *I. ricinus* and *D. marginatus* (Duron et al., 2017) is more surprising, though as these have never previously been isolated in culture, their *in vitro* requirements remain to be determined.

Here we report the first isolation and partial phylogenetic characterisation of *Spiroplasma* spp. from ticks originating from The Netherlands, Poland and Spain. Tick-borne *Spiroplasma* spp. have been isolated previously from *Haemaphysalis leporispalustris* (*Spiroplasma mirum*, Clark, 1964) and *Ixodes pacificus* (*Spiroplasma ixodetis*, Tully et al., 1977; Yunker et al., 1987) in the western United States, from an unspecified *Ixodes* sp. tick in Germany (*Spiroplasma* sp., Henning et al., 2006) and from *I. ricinus* ticks collected in Slovakia (*Spiroplasma* sp. [Bratislava], Bell-Sakyi et al., 2015). The morphology of our novel *Spiroplasma* isolates determined by light and electron microscopy resembled that of previously-studied tick-borne *Spiroplasma* spp. (Tully et al., 1977, 1995; Henning et al., 2006; Bell-Sakyi et al., 2015), although we did not observe the unique 8-nm-thick sub-plasmalemmal structure reported by Tully et al. (1995) in axenically-cultured *S. ixodetis*. Additionally, *Spiroplasma* have been detected by molecular analysis in European *I. ricinus* (Palomar et al., 2016; Subramanian et al., 2012; Tveten and Sjastad, 2011) and in Japanese *Ixodes ovatus* (Qiu et al., 2014; Taroura et al., 2005). Hornok et al. (2010) detected *Spiroplasma* spp. in 5/94 female and 1/9 nymphal *I. ricinus* and 3/23 male and 1/34 female *D. marginatus* unfed tick pools from Hungary. A recent study in Czech Republic (Klubal et al., 2016) reported prevalence of 5% for *Spiroplasma* in pools of 1–7 *I. ricinus* ticks collected from animal hosts; 16S rRNA sequence analysis revealed two clusters, one close to *S. mirum* associated with ticks from dogs and the other close to *Spiroplasma melliferum* (of honeybees) associated with ticks from cats. *Spiroplasma* has not previously been reported from *D. reticulatus*. In the present study, we isolated *Spiroplasma* in cultures derived from 2/12 *I. ricinus* egg batches, 1/6 *D. marginatus* egg batches, 3/14 *D. reticulatus* egg batches and 1/10 unfed adult *D. reticulatus* ticks, suggesting that the overall prevalence of *Spiroplasma* spp. in European ticks is quite high. As previously discussed, the medical and veterinary significance of tick-borne spiroplasmas is unclear (Bell-Sakyi et al., 2015); since then, two additional human cases of *Spiroplasma* infection have been reported, one of which was associated with arthropod stings (Etienne et al., 2018; Mueller et al., 2015), suggesting that further research in this field is warranted.

Ticks have been known to harbour *Rickettsia* spp. for almost 100 years (Cowdry, 1925) and numerous species and strains isolated into vertebrate cells are readily available. Until recently, isolation from infected ticks and other arthropods into tick cells has rarely been reported, probably due more to the small number of laboratories holding tick cell lines and concurrently having access to infected samples rather than the inability of the *Rickettsia* to infect and grow in such cell lines. Simser et al. (2002) described isolation of *Rickettsia monacensis* from internal organs of an *I. ricinus* tick co-cultured with ISE6 cells. In the absence of cell lines derived from fleas, Pornwiroon et al. (2006) and Thepparit et al. (2011) used the ISE6 cell line to isolate *Rickettsia felis* from, respectively, homogenised cat fleas *Ctenocephalides felis* and the common booklouse *Liposcelis bostrychophila*. Baldridge et al. (2010) mentioned isolation of *Rickettsia amblyommatis*, previously known as *Rickettsia amblyommii* (Karpathy et al., 2016), from *Amblyomma* spp. ticks into ISE6 cells and a rickettsial symbiont of *I. scapularis* into IRE11 cells. *R. amblyommatis* was isolated from *Amblyomma americanum* ticks into ISE6 and *A. americanum* AAE2 cells by Sayler et al. (2014). Santibáñez et al. (2015) and Wijnfeld et al. (2016) reported isolation and propagation of *R. raoultii* from homogenised adult ticks, specifically *D. marginatus* in a *Rhipicephalus sanguineus* sensu lato cell line and *D. reticulatus* in the BME/CTVM2 cell line respectively. Kurtti et al. (2015) isolated a novel endosymbiont of *I. scapularis*, *Rickettsia buchneri*, into IRE11 and ISE6 cells. Isolation of *Rickettsia* spp. in primary cell cultures or cell lines derived from naturally-infected embryonic ticks has only been reported on four occasions – *Rickettsia peacockii* in the *Dermacentor andersoni* cell line DAE100 (Simser et al., 2001), *Rickettsia hoogstraalii* in the *Carios capensis* cell line CCE3 (Mattila et al., 2007), *Candidatus* Rickettsia andeanae in primary embryo-derived *Amblyomma maculatum* cell cultures (Ferrari et al., 2013) and *R. raoultii* in primary embryo-derived *D. reticulatus* cell cultures (Alberdi et al., 2012a). Our study has confirmed the ease with which *Rickettsia* spp. can be isolated into tick cell lines following inoculation of organs from potentially-infected field ticks (1/1 *D. marginatus* and 5/10 *D. reticulatus*) and from embryo-derived cell cultures (5/5 *D. marginatus*), as well as the high levels of *Rickettsia* infection reported in *Dermacentor* spp. ticks in some previous studies in Spain and Poland (Márquez et al., 2006; Oteo et al., 2006; Stanczak et al., 2018).

Few previous studies have associated mycobacteria with ticks. A possible role for ticks in leprosy epidemiology has long been suspected in Brazil (de Souja-Araujo and Miranda, 1942). Persistence for 15 days of the causative agent *Mycobacterium leprae* inside midgut cells was demonstrated in experimentally-infected *Amblyomma cajennense* sensu lato ticks (Ferreira et al., 2013). Very recently, transovarial transmission of *M. leprae* in *Amblyomma sculptum* ticks and replication of *M. leprae in vitro* in a tick cell line has been described (Ferreira et al., 2018). Egyed and Makrai (2013) reported isolation on blood agar of *M. chelonae* and *Mycobacterium franklinii* from questing adult female *I. ricinus* (1/8 ticks positive) and nymphal *Haemaphysalis concinna* (1/16 ticks positive) in Hungary. These ticks were homogenised after soaking in 1% formaldehyde for an hour to inactivate bacteria on the external surface, and the authors therefore maintained that the mycobacteria were isolated from inside the ticks’ bodies. Our observation, that a *Mycobacterium* sp. was isolated from dissected internal organs of a tick previously surface-sterilised by a protocol regularly used successfully in preparation of long-lived *in vitro* cultures of tick cells and tissues (Bell-Sakyi, 1991; Bell-Sakyi et al., 2009), supports the view that the mycobacteria originated from inside the tick, rather than from the external surface. Further study is needed to determine whether the putative biofilm-forming microorganism isolated alongside the *Mycobacterium* sp. is a valid microorganism.

Phylogenetic analyses of gene sequences from the newly-isolated *Spiroplasma* strains and sequences published in GenBank revealed that, despite originating from three different tick species in three different countries (The Netherlands, Poland and Spain), they were all closely related to each other, to the Bratislava 1 strain previously isolated from Slovakian *I. ricinus* (Bell-Sakyi et al., 2015) and to the validated species *S. ixodetis*. The number of gene fragments (16S rRNA, *hsp65, sodA* and *rpoB*) from the *Mycobacterium* sp. isolated in the present study are insufficient to confirm its identity as a novel *Mycobacterium* species but show that it belongs to the *M. chelonae* complex of fast-growing, free-living mycobacteria. As such, it is possible that it was an environmental contaminant ingested by the tick during feeding on its canine host, rather than a true tick endosymbiont; *M. chelonae* and other fast-growing mycobacteria have previously been isolated from the skin of dogs and cats (Jang and Hirsch, 2002; Govendir et al., 2011). However, in view of the successful transovarial transmission of *M. leprae* by *A. sculptum* reported by Ferreira et al. (2018), we cannot rule out the possibility that ticks may occasionally harbour endosymbiotic mycobacteria.

In conclusion, this study confirms the high prevalence and wide diversity of bacterial species associated with *Ixodes* and *Dermacentor* spp. ticks collected in different locations across Europe. Ixodid tick cell lines proved to be sensitive and effective, though in some cases rather slow, systems for detection and isolation of fastidious bacteria both from tick embryos and from organs dissected from unfed, partially-fed and fully-engorged adult ticks. Although it was unclear if failure to isolate bacteria from tick organs in the present study was due to absence of bacteria in the inoculum, it was evident that some tick cell lines were more susceptible to infection with particular bacterial species than others. Differences between cell lines in susceptibility to, and intensity of, infection could be used to study the molecular basis of the host range of particular bacterial species. Cell culture isolation increases confidence that a bacterium detectable by molecular methods is located inside the tick, rather than merely a surface contaminant. In particular, isolation from embryos increases the likelihood that the bacterium is a true endosymbiont, rather than being a passenger in the previous blood meal derived from a vertebrate host. These techniques, combined with subsequent molecular analysis, will greatly aid understanding of tick-bacteria relationships.

## Funding

The study was funded by the UK Biotechnology and Biological Sciences Research Council (grant numbers BBS/E/1/00001741 and BB/P024270/1). AMP was partially supported by the Fundación Rioja Salud (grant: FRS/PIF-01/10) and the ‘Sociedad Española de Enfermedades Infecciosas y Microbiología Clínica’ (grant: ‘Becas de estancia en el extranjero’). The NGS was performed under the project PI15/02269 supported by “Fondo de Investigaciones Sanitarias (Acción Estratégica en Salud 2015, ISCIII, M. Economía y Competitividad (Spain)”.
